# *Hylocereus polyrhizus* Pulp Residues Polysaccharide Alleviates High-Fat Diet-Induced Obesity by Modulating Intestinal Mucus Secretion and Glycosylation

**DOI:** 10.3390/foods14152708

**Published:** 2025-08-01

**Authors:** Guanghui Li, Kit-Leong Cheong, Yunhua He, Ahluk Liew, Jiaxuan Huang, Chen Huang, Saiyi Zhong, Malairaj Sathuvan

**Affiliations:** 1 Guangdong Provincial Key Laboratory of Aquatic Product Processing and Safety, College of Food Science and Technology, Guangdong Ocean University, Zhanjiang 524088, China; lgh15967594856@126.com (G.L.); iheyunhua@gmail.com (Y.H.); huangjiaxuan11@stu.gdou.edu.cn (J.H.); zhongsy@gdou.edu.cn (S.Z.); 2 Guangdong Province Engineering Laboratory for Marine Biological Products, Zhanjiang 524088, China; 3 Guangdong Provincial Engineering Technology Research Center of Seafood, Zhanjiang 524088, China; 4 Guangdong Provincial Engineering Technology Research Center of Prefabricated Seafood Processing and Quality Control, Zhanjiang 524088, China; 5 Collaborative Innovation Center of Seafood Deep Processing, Dalian Polytechnic University, Dalian 116034, China; 6 Guangdong Meichen Biotechnology Co., Ltd., Guangdong Suixi Dragon Fruit Science and Technology Small Courtyard, Zhanjiang 524088, China; ahlukliew@163.com; 7 Dr. Neher’s Biophysics Laboratory for Innovative Drug Discovery, State Key Laboratory of Quality Research in Chinese Medicine, Macau University of Science and Technology, Taipa, Macao SAR 999078, China; chuang@must.edu.mo; 8 Center for Advanced Studies in Botany, University of Madras, Chennai 600025, India; sathuvansjc@gmail.com

**Keywords:** *Hylocereus polyrhizus* pulp residues polysaccharide, Metabolic syndrome, antioxidant activity, O-glycopeptide, site-specific o-glycoproteomics

## Abstract

Although *Hylocereus polyrhizus* pulp residues polysaccharides (HPPP) have shown potential in improving metabolic disorders and intestinal barrier function, the mechanism by which they exert their effects through regulating O-glycosylation modifications in the mucus layer remains unclear. Therefore, this study established a HFD-induced obese colitis mouse model (*n* = 5 per group) and combined nano-capillary liquid chromatography-tandem mass spectrometry (nanoLC-MS/MS) technology to quantitatively analyze the dynamic changes in O-glycosylation. Additionally, through quantitative O-glycosylation proteomics and whole-proteome analysis, we identified 155 specifically altered O-glycosylation sites in colon tissue, with the glycosylation modification level of the MUC2 core protein increased by approximately 2.1-fold. The results indicate that HPPP alleviates colonic mucosal damage by regulating interactions between mucus O-glycosylation. Overall, we demonstrated that HPPP increases HFD-induced O-glycosylation sites, improves intestinal mucosal structure in obese mice, and provides protective effects against obesity-induced intestinal mucosal damage.

## 1. Introduction

Metabolic syndrome (MetS) is a complex chronic disease characterized by abdominal obesity, insulin resistance, dyslipidemia, hypertension and hyperglycemia. Notably, obesity is not only one of the core features of MetS, but also a key risk factor for its development. Obesity can significantly exacerbate the condition of MetS and increase the difficulty of treatment, and it is also a direct cause of many metabolic diseases, such as type 2 diabetes mellitus, nonalcoholic fatty liver disease, and atherosclerotic cardiovascular disease [1,2,3,4]. and obesity-associated metabolic disorders (e.g., lipid and glucose me-tabolism), as well as accompanying chronic inflammation and hormonal changes [5,6], constitute the main factors closely associated with MetS and its complications and its complications. A growing number of studies have shown that the pathogenesis of obesity and MetS is mainly due to the complex interplay of genetics, environment, lifestyle habits, drugs, and diseases [7,8]. However, patients with MetS are often associated with dysfunction of the intestinal mucus layer, and this injury can lead to increased intestinal permeability, allowing intestinal microbes and their metabolites (e.g., endotoxins) to leak into the portal circulation and enter the liver [9,10,11]. These microbial metabolites may further trigger immune stimulation and circulatory disorders, thereby exacerbating the risk of obesity and related metabolic disorders [12]. In addition, dietary polysaccharides have been shown to influence mucin O-glycoprotein chain expression by modulating gut microbes and their metabolites [13].

Notably, MetS is closely associated with intestinal mucus barrier dysfunction, whose core component is the highly glycosylated mucin MUC2 (accounting for 80% of molecular weight) secreted by goblet cells [14,15,16]. MUC2 forms homodimers in the endoplasmic reticulum and undergoes glycosylation in the Golgi apparatus to establish the protective mucus layer. Impairment of this barrier leads to increased intestinal permeability, facilitating microbial translocation to the portal circulation and liver, thereby exacerbating obesity and metabolic disorders Both high- HFD and high fructose intake disrupt MUC2 expression and secretion, resulting in mucus layer thinning and metabolic deteriorationWhile MUC2 glycosylation plays crucial roles in maintaining barrier function and modulating gut microbiota composition [17,18]. its direct mechanisms in preventing obesity and MetS progression require further investigation. With the increasing industrialized processing of *Hylocereus polyrhizus* (dragon fruit), such as processing into juice, wine, jam, preserves and other products, a large amount of by-products—*Hylocereus polyrhizus* pomace, which mainly consists of peel, seeds and pulp, usually accounts for 30–50% of the total weight of the processed fruit, will be produced [19]. Data show that *Hylocereus polyrhizus* pomace contains a variety of bioactive substances, such as plant polysaccharides, vitamin C and flavonoids, etc., of which HPPP have attracted much attention because of their unique structure and multifunctional properties [20]. Studies have shown that HPPP have important roles in regulating glucose and lipid metabolism, which makes them potential intervention targets for obesity and metabolic disorders [21]. Specifically, HPPP significantly attenuated weight gain, reduced fat accumulation, regulated blood glucose and lipid levels, and also enhanced hepatic antioxidant capacity in HFD-induced obese mice. In addition, HPPP showed an important role in intestinal barrier function, as their high content of mucus glycosylation helped to maintain mucus stability and had a protective and reparative effect on intestinal barrier damage. These studies suggest that HPPP may have multiple regulatory mechanisms or different functions in different tissues, but its function in metabolic diseases remains unclear.

This study focuses on the effects of HPPP on a diet-induced obese mouse model. Specifically, this study explores the beneficial effects of natural polysaccharides on obesity-associated mucosal injury and their underlying mechanisms with respect to intestinal barrier function, cuprocyte differentiation, mucus production and secretion, and transmembrane mucin glycosylation. In addition, this study employed quantita-tive O-glycan proteomics and global proteomics approaches to identify specific O-glycan sites in colonic tissues. These findings are significant and can reveal the role of HPPP in ameliorating HFD-induced associated mucosal injury and dysregulation of colonic mucin O-glycans, thus providing a new scientific basis and target of action for dietary intervention in MetS and related metabolic disorders.

## 2. Materials and Methods

### 2.1. Chemicals and Materials

*Hylocereus polyrhizus* pomace was provided by Meichen Group Co., Ltd. (Zhan-jiang, China), It is a by-product of the cold-pressed juice extraction process, where fresh fruits were mechanically pressed to separate the juice, and the remaining pomace (containing peel, pulp fibers and seeds) was collected. The following analytical grade chemicals were used: hydrochloric acid (HCl, 36–38%, Sinopharm Chemical Reagent Co., Ltd., Shanghai, China), ethanol (C_2_H_5_OH, ≥99.7%, Shanghai Aladdin Biochemical Technology Co., Ltd., Shanghai, China), and 1,1-dimethylbiguanide hydrochloride (metformin, ≥98%, Solarbio Science & Technology Co., Ltd., Beijing, China; Catalog No.: M8090). All other chemicals were of analytical grade or higher purity.

### 2.2. Extraction of HPPP

The extraction method of HPPP was referred to the previous studies and appropriately modified as follows [22]: Firstly, the filtrate residue from the industrialized production of *Hylocereus polyrhizus* was dried, crushed and sieved to obtain *Hylocereus polyrhizus* pomace powder. Then, 50 g of pomace powder was weighed and mixed with deionized water in the ratio of 1:10 (*w*/*v*), and then the pH was adjusted to 2 with hydrochloric acid, followed by heating in a water bath environment at 90 °C for 2 h. At the end of the reaction, it was first concentrated using spinning, Four volumes of ice-cold 95% ethanol (4:1 *v/v* relative to the concentrated extract) were added dropwise under constant stirring at 200 rpm, and left overnight at 4 °C to induce precipitation. Afterwards, the supernatant was collected by centrifugation at 1789× *g* for 5 min. Finally, the sample was dialyzed for 24 h using a dialysis bag with a molecular weight cut-off of 500 Da to remove impurities. The dialysed sample was then frozen at −80 °C for 4 h and freeze-dried in an LGJ-10 freeze dryer (Beijing Songyuan Huaxing Technology Development Co., Ltd., Beijing, China) under the following conditions: condenser temperature −50 °C, vacuum pressure 0.120 mBar, with a final 6-h secondary drying at 25 °C to obtain the final HPPP.

We optimized the HPPP extraction process for *Hylocereus polyrhizus* fruit pulp to ensure batch-to-batch consistency and implemented detailed quality control measures at critical steps. The raw material was standardized using 80-mesh sieved powder (CV = 4.2%, *n* = 5 batches) from the same cultivation batch. Pilot extraction (*n* = 5) established reproducible yields, with 2.2 ± 0.5 g of HPPP extracted per 50 g of fruit pulp (efficiency of 4.4% ± 1.1%). Key process parameters were rigorously validated: (1) Hydrolysis at pH 2.0 maximizes polysaccharide recovery (23% higher than the pH 1.5–3.0 range, verified by HPLC) while minimizing protein degradation; (2) 4-fold volume 95% ethanol precipitation achieves optimal 89% purity, superior to other ratios. Post-extraction quality control confirmed: carbohydrate content ≥16% (phenol-sulfuric acid method), protein content ≤ 5% (Coomassie Brilliant Blue method), and inter-batch FTIR spectrum consistency > 95%.

### 2.3. Determination of Chemical Composition

The chemical composition of HPPP was analyzed using validated methods: (1) Total sugar content was determined by the phenol-sulfuric acid method [23] using D-glucose (Sigma-Aldrich, St. Louis, MO, USA, G8270) as the standard (0–100 μg/mL calibration curve, R^2^ > 0.998), with samples (50 μL) reacted with 5% phenol (150 μL) and concentrated H_2_SO_4_ (750 μL) followed by 30 min incubation at 30 ± 1 °C and A_490_ measurement (inter-assay CV = 3.8%); (2) Glucuronic acid content was measured via the m-hydroxybiphenyl method [24] with D-galacturonic acid standard (Sigma, G0501), involving borate-sulfate pretreatment and A_520_ measurement after 20 min color development; (3) Protein content was quantified using the Coomassie Brilliant Blue method [25] with BSA standard (Thermo Fisher, 23209), including TCA precipitation to remove phenolics prior to A_595_ measurement. All assays were performed in triplicate with appropriate blanks

### 2.4. X Diffraction Analysis

The crystalline zone of HPPP was analyzed by X-ray diffraction. The measurement range was 5~50° (2θ) with a step size of 0.02° and a counting time of 1 s/step.

### 2.5. Thermogravimetric Analysis

The thermal properties of HPPP were investigated using a thermo-synchronous analyzer [26], 5.0 mg of polysaccharide samples were placed in an alumina crucible and an empty alumina crucible was used as a blank control, the experimental atmosphere was nitrogen and the samples were heated up from 30 °C to 800 °C at a rate of 10 °C/min to determine the mass change of HPPP.

### 2.6. Rheological Property Measurements

Referring to the method of Li et al. [27] with some modifications. Rheological properties were characterized using a HAAKE MARS 60 rheometer (Thermo Fisher Scientific, Karlsruhe, Germany) equipped with a 35 mm diameter parallel plate geometry (PP35 Ti, titanium alloy). HPPP solutions (0.1%, 0.5%, 1.0% *w*/*v*, using deionized water as the solvent) and a 1% sodium carboxymethyl cellulose (CMC, Sigma-Aldrich, 419273) control group were prepared 24 h prior to testing to ensure complete hydration. The viscosity profiles of HPPP were determined at 25 °C when the shear rate of HPPP was varied from 0.1 s^−1^ to 1000 s^−1^.

### 2.7. Antioxidant

#### 2.7.1. Determination of DPPH Radical Scavenging Capacity

The DPPH-scavenging rate of HPPP was determined according to the method of Su et al. [28]. 2.0 mL of different concentrations of the sample solution (0.3125, 0.625, 1.25, 2.5, 5.0 mg/mL) were taken into centrifugal tubes, and 2.0 mL of the DPPH-ethanol solution (0.04 mg/DPPH-ethanol solution (0.04 mg/mL) was added into the centrifuge tubes, and the reaction was carried out in dark environment for 30 min. The absorbance at 517 nm was measured by an enzyme counter, and the absorbance of ethanol solution was used as a blank (Ao), and ascorbic acid was used as a positive control.

$$\mathrm{DPPH}-\mathrm{clearance}\;(\%)=\left[1-\frac{A1-A2}{A0}\right]\times 100\%$$

where: A_1_ is the absorbance of DPPH solution added to the test solution: A_2_ is the absorbance of DPPH solution added to the test solution; A_0_ is the absorbance of DPPH solution added to water.

#### 2.7.2. Scavenging Assay of Free Radical Scavenging Ability of ABTS

Weigh 200.0 mg of ABTS and 34.4 mg of potassium persulfate, dissolve in 50.0 mL of distilled water, shake well, and leave it at room temperature and away from light for 24 h, as the mother liquor of ABTS. Take an appropriate amount of ABTS mother liquor, diluted with 95% ethanol until the absorbance value was within 0.70 ± 0.02 (OD_734_), as the ABTS measurement solution, the solution should be prepared and used now. Different concentrations (0.3125, 0.625, 1.25, 2.5, 5.0 mg/mL) of HPPP were added to the ABTS working solution at 1:9, fully homogenized, and the absorbance value was measured at 734 nm after 6 min reaction protected from light, and the scavenging activity of the specimen against ABTS radicals was calculated by the following formula.

$$\mathrm{ABTS}-\mathrm{clearance}\;(\%)=\left[1-\frac{A1-A2}{A0}\right]\times 100\%$$

where: A_1_ is the absorbance of ABTS solution added to the test solution: A_2_ is the absorbance of ethanol added to the test solution; A_0_ is the absorbance of water added to the ABTS solution.

### 2.8. Animal Experiments

Male C57BL/6 mice (25 ± 2 g, *n* = 5/group) were purchased from Zhuhai Biotest Technology Co., Ltd. (Zhuhai, Guangdong, China). The Animal Care and Welfare Committee of Guangdong Ocean University (GDOU-LAE-2024-011) approved all animal experimental protocols, and the experiments were conducted in accordance with the Guide for the Care and Use of Laboratory Animals. Mice were housed in the animal facilities of Guangdong Ocean University (Experimental Animal Use License: SYXK (YUE)-20240204) at an ambient temperature of 25 ± 2 °C, humidity of 50 ± 10%, and a light/dark cycle of 12 h. Mice were allowed to eat and drink freely. The length of the experiment was 11 weeks, and all mice had 1 week to acclimatize to the new environment before the official experiment started. After the acclimatization period, mice were induced to become obese using a 60% HFD. Subsequently, mice were randomly assigned to four groups (*n* = 5 per group) based on body weight (±1 g) using computer-generated randomization: (1) normal diet + saline gavage (NC), (2) HFD+ saline gavage (HFD), (3) HFD + 150 mg/kg dimethylbiguanide hydrochloride (PC), and (4) HFD + 150 mg/kg HPPP. Blinded procedures were implemented throughout the experiment: diet preparation and gavage administration were performed by independent personnel unaware of the group assignments, while outcome assessments (body weight, food intake) were recorded by experimenters unaware of the treatment groups using coded cages. The experimental HFD contained 200 g/kg casein, 3 g/kg L-cysteine, 125 g/kg maltodextrin, 72.8 g/kg sucrose, 50 g/kg cellulose, 25 g/kg soybean oil, and 245 g/kg lard as the primary fat source, supplemented with 50 g/kg mineral mix (S10026B), 1 g/kg vitamin mix (V10001C), and 2 g/kg choline bitartrate. The diet provided 5.22 kcal/g with macronutrient energy distribution of 60% fat (primarily from lard), 20% protein, and 20% carbohydrates. In contrast, the control chow diet contained 506.2 g/kg corn starch instead of maltodextrin, only 20 g/kg lard, and had significantly lower energy density (3.85 kcal/g) with 10% fat, 20% protein, and 70% carbohydrate kcal distribution. Both diets were matched for micronutrient and fiber content to isolate the effects of dietary fat manipulation.

### 2.9. O-Glycopeptide Enrichment and Extraction

The colon tissues were cut into small sections and sent to Suzhou Hanno Biological Co. for testing. First, to enrich and extract O-glycopeptides from the tissues, we used the O-conjugated glycopeptide (EXoO) extraction method for O-glycopeptides from tissues as described by the previous authors [29,30]. First, tissues were disrupted and denatured in 8 M urea/200 mM Tris-HCl (pH 8.0) solution by sonication. Subsequently, proteins were reduced with 5 mM DTT for 1 h at 37 °C and alkylated with 10 mM iodoacetamide for 40 min at room temperature. Next, the samples were diluted and digested with trypsin to generate peptides, which were then guanidinized and desalted by a Sep-Pak C18 column. For overall proteomic analysis, peptide aliquots were frozen and dried by vacuum centrifugation. Afterwards, peptides were subjected to glycopeptide enrichment and hydrophilic enrichment using HyperSep Retain AX columns, and impurities were removed by solid-phase AminoLink Plus coupling resin. Recombinant OgpA and sialidase were expressed and purified as described previously [31]. Subsequently, O-GalNAc glycopeptides were released using OgpA and sialic acid was removed using sialidase, and the glycopeptides were finally desalted, eluted, dried, and resuspended in 0.1% TFA for subsequent analysis.

### 2.10. TMT Labeling and Graded Separation of O-Glycopeptides

Peptides were labeled with (ThermoFisher Scientific) TMT-10-plex according to the manufacturer’s instructions, allowing simultaneous identification and quantification of peptides and 0-glycopeptides. Briefly, peptides were re-solubilized in 0.1 M tetraethylammonium tetrahydroborate buffer, equilibrated to room temperature with the TMT-10-plex labeling reagent immediately prior to use, and each vial of the reagent was dissolved in anhydrous acetonitrile (ACN) and added separately to the sample peptides, which were incubated for 1 h at room temperature. Labeling efficiency was checked by desalting using C18 and analyzing on an Orbitrap Fusion Lumos Tribrid mass spectrometer (Thermo Fisher Scientific). After verification of labeling efficiency, samples were quenched by addition of 5% hydroxylamine and combined. Dry in a SpeedVac evaporator. Pellets were resuspended with 500 µL of 0.1% trans fatty acids and then desalted on the same C18 (Phenomenex, Torrance, CA, USA, 15 μm, 300 Å) SPE column as above. The zic-HILIC column (Merck Millipore, Burlington, MA, USA, 5 μm, 200 Å) was equilibrated with 80% ACN/5% Trifluoroacetic acid (TFA). Load the peptide solution and flush with 80% ACN/5% TFA. Intact glycopeptides were sequentially eluted with 100 µL of 0.1% TFA and 50 mM ABC; eluates were combined. The mixed eluates were dried in a SpeedVac evaporator and resuspended in ultrapure water. Peptide concentrations were determined using a BCA kit. Labeled samples were fractionated by acidic pH reversed-phase chromatography. The mobile phase A was a mixture of 99.9% H_2_O and 0.1% Formic acid and the mobile phase B was a mixture of 99.9% ACN and 0.1% Formic acid. The TMT-10-plex labeled glycopeptides were separated using a gradient at a flow rate of 0.3 mL/min.

### 2.11. RPLC-MS/MS Analysis of Intact O-Glycopeptides

The analysis was carried out on a Dionex Ultimate 3000 RSLC nano HPLC (Thermo Fisher Scientific) using a 70 cm long, 75 μm ID C18 analytical column, where buffer A was a 0.1% formic acid aqueous solution and buffer B was a 0.1% formic acid acetonitrile solution [32,33]. The eluted intact O-glycopeptides were detected by a Q Exactive mass spectrometer with a mass spectral acquisition range of 500–2000 *m*/*z* and a mass resolution of 60,000 (at *m*/*z* 200), and 30,000 in MS/MS mode. Data-dependent mode (Top20) and high-energy collisional dissociation (HCD) were used to acquire fragmentation information, and MS scans were performed by an automatic gain control (AGC) target value and maximum injection time were 3 × 10^6^ and 20 ms, respectively, and the target value and maximum injection time for MS/MS scans were 5 × 10^5^ and 250 ms, respectively. the separation window and dynamic exclusion time were set to 0.7 *m*/*z* and 30.0 s, respectively, and the temperature of the ion-transfer capillary was set to 300 °C, and the spray voltage was set to 1.9 kV.

### 2.12. Identification and Quantification of Intact O-Glycopeptides

O-glycosylation of extracted proteins from mouse colon tissue-derived samples was analyzed by the mass spectrometry-based O-glycoproteomics and O-glycopeptide database search engine pGlyco3.0 [34]. In the database search, Carbamidomethylation and TMT were set as static modifications; intact O-glycopeptide spectrum matches (GPSMs) obtained by false-positive FDR ≤ 1% control were analyzed according to the modifications on the polypeptide backbone (Mod), polypeptide backbone amino acid sequence (Peptide), and polysaccharide linkage structure (PlausibleStruct), and screened out GPSMs containing glycosylation sites scored with glycan chain structure as the final intact O-glycopeptide IDs (Identifications). Visualizations were viewed and exported in pGlyco, including peptide backbone graphical dissociation plots with matching fragment ion annotations and secondary mass spectrometry plots with matching fragment ion annotations. The sample was also searched and matched using the peptide database search engine Proteome Discoverer 2.4 (PD), with static modifications of Alkylation (Carbamidomethyl), TMT, and dynamic modifications of Oxidation, HexNAc, and Acetyl, and the data were processed to obtain qualitative and quantitative results of proteins and peptides [35].

### 2.13. Statistical Analysis

All data are presented as mean ± standard error of the mean (mean ± SEM), de-rived from at least three independent biological replicates with each biological repli-cate measured in duplicate or triplicate (technical replicates). Results obtained from the control and experimental groups were compared by analysis of variance (ANOVA) and Bonferroni multiple comparisons. All statistical analyses were performed by GraphPad Prism 9 (San Diego, CA, USA) and Microsoft Excel 2021.

## 3. Results and Discussion

### 3.1. The Yield and Basic Composition of Polysaccharides from Hylocereus Polyrhizus Pomace

The dry weight extraction rate of active polysaccharides from *Hylocereus polyrhizus* pomace using acid extraction and alcohol precipitation was about 4.4 ± 0.8%, and the analysis of the fractions showed that the total sugar content was 16.43%, of which the content of galacturonic acid was 10.39% (Table 1). This significantly high content of galacturonic acid clearly reflects that the crude extract is rich in pectin-like polysaccharides. The main component of pectin is galacturonic acid, and its content is one of the important indicators of pectin quality [36]. This result not only confirms the efficient enrichment of negatively charged glucuronic acid groups by the acid extraction and alcohol precipitation process, but also strongly suggests that the polysaccharides contained therein have good bioactive potentials, because glucuronic acid is recognized as a key structural basis for plant polysaccharides to exert immunomodulatory and antioxidant effects [37,38]. In addition, the coexistence of trace proteins (3.51%) suggests the possibility of protein impurity co-precipitation or, more notably, the existence of natural glycoprotein complexes, which have been demonstrated in several studies to produce significant synergistic effects through sugar-protein interactions that can further influence or even enhance polysaccharide functions [39].

### 3.2. X-Ray Diffraction Characterization of HPPP

**Figure 1:**
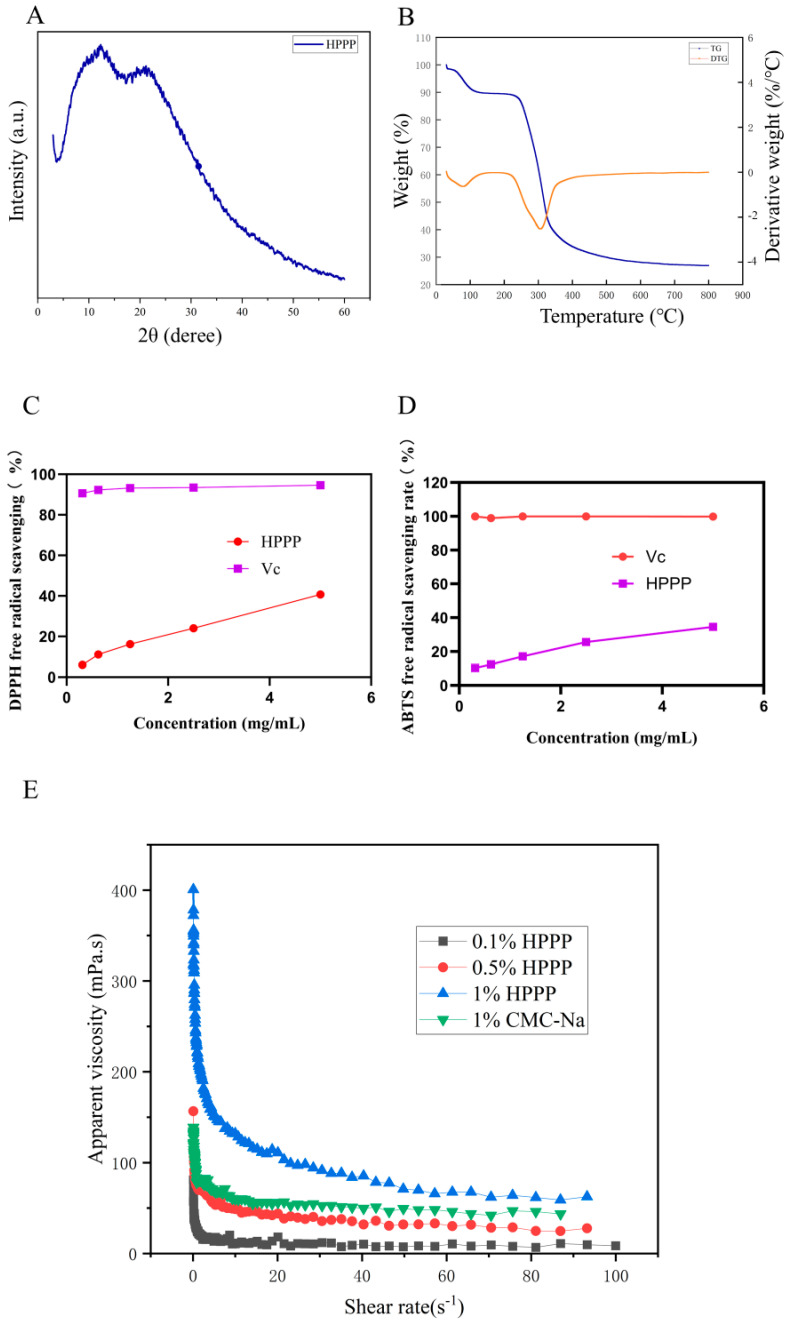
Physicochemical properties of HPPP. (**A**) X-diffraction pattern of HPPP; (**B**) Thermogravimetric analysis of HPPP; (**C**) Viscosity of HPPP solutions as a function of shear rate; (**D**) DPPH radical scavenging activity; (**E**) ABTS radical scavenging activity.

The crystal structure of HPPP was analyzed by XRD (Figure 1A). There are broad diffuse peaks between 5° and 50°, and no sharp diffraction peaks. The HPPP sample may be predominantly amorphous, and structurally it is a typical amorphous polymer. Amorphous polymers have an elastic structure [40].

### 3.3. Thermal Cleavage Behavior of HPPP

Thermal stability is an important physicochemical property of polysaccharides in the food industry. TG and DTG are usually used to evaluate the thermal properties of polysaccharides. From the thermogravimetric analysis curve (Figure 1B), it can be seen that dragon fruit pomace polysaccharide has three obvious mass changes during the high temperature process. The first weight loss occurred at 30~220 °C, which may be due to the heat evaporated the water in polysaccharides, which is mainly bound to solids such as polysaccharides and proteins through hydrogen bonding, and the weight loss of polysaccharides at this stage was 17.86%, and the second weight loss occurred at 220~340 °C, which may be due to the degradation of the long chains of carbohydrates and the aggregation of fragments [41], and the weight loss of polysaccharides was 69.94% in this part. weight loss was 69.94%, and the maximum weight loss was at 300 °C. The third weight loss occurred after 340 °C, and the mass loss was relatively slowed down, which could be attributed to the fact that although the polysaccharides were further decomposing, the weight loss was slowed down due to the fact that the remaining polysaccharides were more thermally stable such as some residual cellulose or most of the polysaccharides had been carbonized [42].

### 3.4. Rheological Behavior of HPPP Solutions

The apparent viscosity of HPPP solutions varied with shear rate (Figure 1C). With the increasing concentration of polysaccharide, the apparent viscosity of HPPP was increasing. At the same time, with the increasing shear rate, the apparent viscosity of the polysaccharide solution decreases, and the solution shows the characteristics of shear thinning, which exhibits the characteristics of pseudoplastic fluid. When the polysaccharide concentration was 1.0% (*w*/*v*), its apparent viscosity was close to that of 1.0% (*w*/*v*) sodium carboxymethylcellulose, and the trends of the two curves were nearly the same, which indicated that the polysaccharide solution formed an intertwined network structure in the aqueous phase, which changed the original properties of the aqueous solution, and made the solution show the characteristics of non-Newtonian fluid behavior. This phenomenon is related to the viscoelastic protective layer formed by HPPP on the mucous membrane surface.

### 3.5. Antioxidant Properties

DPPH free radicals are widely used to assess the hydrogen supplying capacity of various antioxidants. In the present study, the scavenging activity of HPPP against DPPH radicals (Figure 1D). The scavenging activity of HPPP against DPPH at different concentrations was lower than that of vitamin C, but the scavenging activity gradually increased with increasing concentration.

The ABTS radical is a stable free radical cation and is commonly used to assess the free radical scavenging ability of antioxidants. Studies have shown that ABTS radicals are closely associated with the development of several oxidative stress-related diseases. Therefore, in this study, the in vitro ABTS radical scavenging activity (Figure 1E) of HPPP was determined. The ABTS radical scavenging rate of the samples increased with increasing polysaccharide concentration, showing a significant dose-dependence.

### 3.6. HPPP Alter O-Glycosylation in the Colonic Mucus Layer

**Figure 2:**
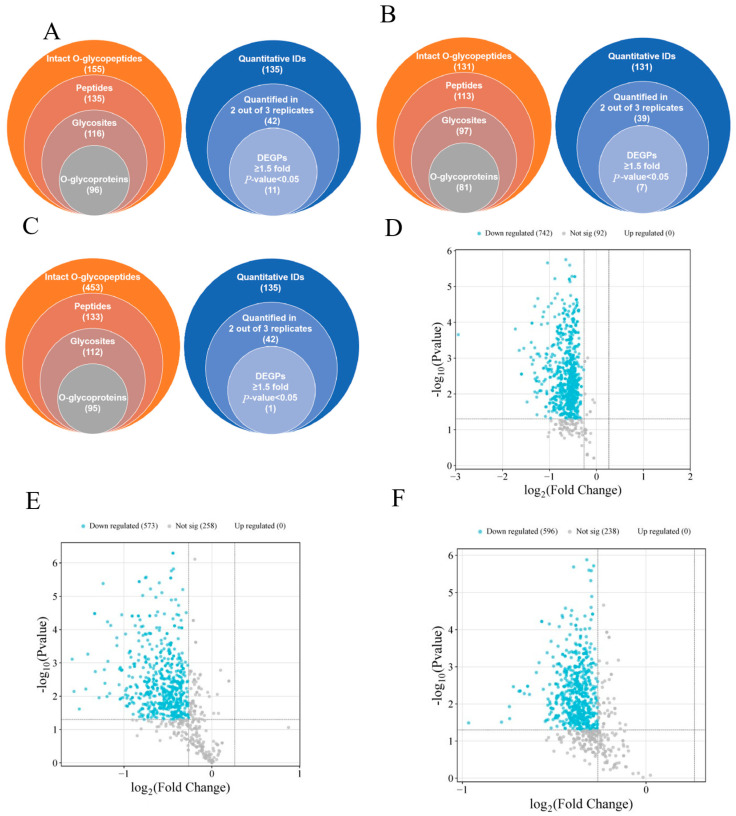
HPPP modulates colonic O-glycosylation. Overall qualitative (left pie) and quantitative results (right pie) of O-glycopeptides from TMT-labeled RPLC-MS/MS analyses of (**A**) stacked Venn diagrams of the qualitative and HFD vs. NC quantitative results for sample NC; (**B**) stacked Venn diagrams of the qualitative and HFD vs. HFD-PC quantitative results for sample HFD-PC; and (**C**) stacked Venn diagrams of the qualitative and HFD vs. HFD-HPPP quantitative results for sample HFD-HPPP quantitative results stacked Venn diagrams. (**D**) Volcano plot of differentially expressed proteins in the HFD vs. NC sample group; (**E**) Volcano plot of differentially expressed proteins in the HFD vs. HFD-PC sample group; (**F**) Volcano plot of differentially expressed proteins in the HFD vs. HFD-HPPP sample group.

It has been shown that O-glycosylation patterns are disturbed when glycosyltransferase activity is inhibited [43]. This abnormality causes the mucus layer to be more susceptible to degradation by proteases secreted by pathogenic bacteria (e.g., Enterobacteriaceae), accelerating barrier breakdown and leading to abnormal O-glycosylation. To investigate the underlying mechanisms in more depth, we analyzed the composition of colonic mucin O-type glycans using RPLC-MS/MS. The intensity of the reporter ions was extracted with pGlycoQuant. In three technical replicates with no less than two observation controls, as obtained by Wayne plots (Figure 2A–C), the HFD-HPPP intervention significantly elevated mouse colonic O-glycopeptide diversity, with 453 qualitatively detected, which was 2.9-fold higher than that of the NC group; whereas the HFD-PC group showed an inhibitory trend. Quantitative analysis showed an intervention-dependent disturbance of glycosylation homeostasis in the HFD group: compared to the NC group, which showed 11 differentially expressed glycoproteins (DEGPs), the PC group mitigated to 7, whereas the HPPP group showed only 1 DEGP, a decrease of 91%. This gradient change confirmed that plant polysaccharides almost completely blocked HFD-induced glycoprotein abnormalities by maintaining O-glycosylation homeostasis, and in addition, by selecting five proteins that had an effect on the mucosa, it was found that four of these O-glycopeptides were down-regulated, and one was up-regulated (Table 2). The corresponding down-regulated O-glycoproteins were Dcn, Fbn1, Lamb1, and Hspg2; the up-regulated O-glycoprotein was Vasp, which provides a molecular basis for targeting glycosylation modifications to improve the intestinal mucosal barrier.

In the HFD versus NC group comparison (Figure 2D), the volcano plot showed significant and widespread protein expression suppression: 742 proteins were significantly down-regulated (blue dots), clustered in a region of high significance (−log10 (*p*-value) > 4), and there were no up-regulated proteins. This indicated that HFD caused systemic protein expression collapse in the NC group, especially affecting the core structure of the mucus barrier; in the comparison between the HFD and HFD-PC groups (Figure 2E), it showed a partial mitigating effect of the positive drug intervention, but there were still no up-regulated proteins, suggesting that PC, although mitigating some of the damage, failed to activate the expression of reparative proteins; in the comparison between the HFD and HPPP groups (Figure 2F), HPPP demonstrated efficient protection, the number of down-regulated proteins was further reduced, and the proportion of undifferentiated proteins was significantly elevated.

### 3.7. Glycoprotein Structural Domains Remodeled by HPPP in Obese Mice

**Figure 3:**
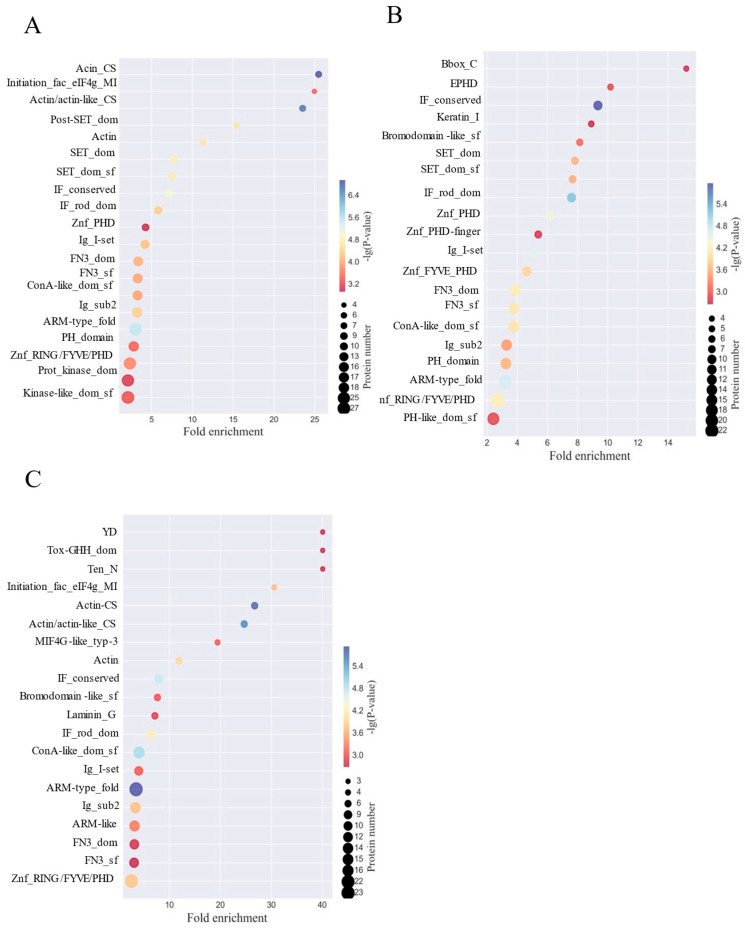
HPPP remodeling glycoprotein structural domains in obese mice. (**A**) Analysis of differentially expressed glycoprotein structural domains in the HFD vs. NC sample group; (**B**) Analysis of differentially expressed glycoprotein structural domains in the HFD vs. HFD-PC sample group; (**C**) Analysis of differentially expressed glycoprotein structural domains in the HFD vs. HFD-HPPP sample group.

In order to reveal the structural domain enrichment characteristics of glycoproteins and to find significantly enriched structural domains and their corresponding glycoproteins by evaluating the significance level of protein enrichment under a certain structural domain entry, structural domain enrichment analysis was performed [44], which showed that HFD disruption mainly targets cytoskeletal and nuclear regulatory structural domains, and that, in the comparison of the HFD versus the NC group (Figure 3A), actin structural domains (Actin/actin-like_CS, 18-fold enriched, protein 25) and transcription start domain (Initiation_fac_eIF4g_MI, 15-fold enriched, protein 25) were significantly activated, accompanied by high expression of nuclear modification domains (SET_dom_sf, Znf_PHD) (12-fold enriched on average), revealing that HFD impairs the cytoskeleton and gene regulation through disruption of skeletal stability and gene regulation to damage the intestinal barrier; whereas the HFD-PC group partially alleviated the skeletal disorder (Figure 3B) In addition, the HFD-HPPP group drove the remodeling of defensive structural domains (Figure 3C), with strong expression of the toxin-responsive domain Tox-GHH_dom (23-fold enriched, Protein 30) and the basement membrane adhesion domain Laminin_G (22-fold enriched), and the simultaneous and complete removal of the HFD associated actin/nuclear domains (Actin/SET class nulling) and activation of the exogenous detoxification domain YD (16-fold enrichment)-confirming that HPPP achieves active intestinal mucosal protection at the molecular level by establishing a “detoxification-adhesion” double barrier. HPPP is a molecularly active defense of the intestinal mucosa by establishing a double barrier of “detoxification-adhesion”.

### 3.8. HPPP Drive Functional Reconfiguration of Glycoproteins for Efficient Intestinal Mucosal Barrier Defense

**Figure 4:**
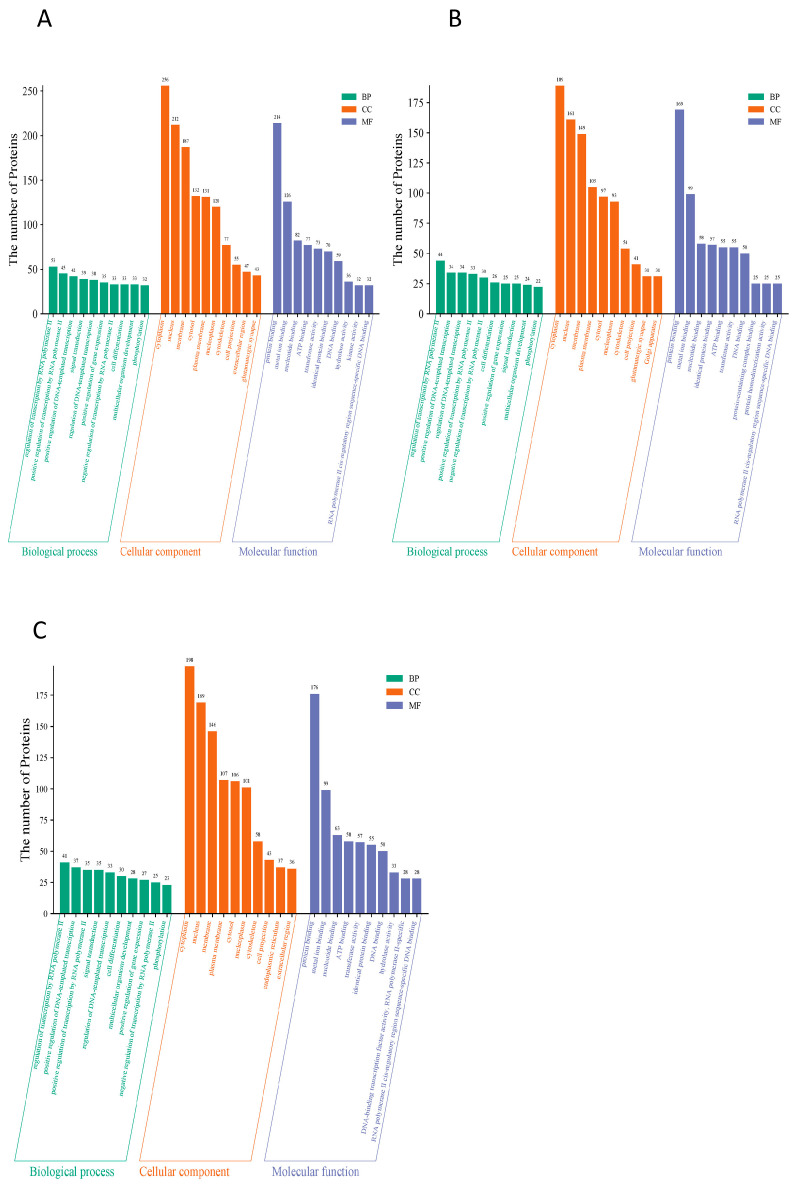
HPPP drives glycoprotein functional remodeling for efficient intestinal mucosal barrier defense. (**A**) Differential expression of glycoprotein GO annotation diagram for HFD vs. NC sample group; (**B**) Differential expression of glycoprotein GO annotation diagram for HFD vs. HFD-PC sample group; (**C**) Differential expression of glycoprotein GO annotation diagram for HFD vs. HFD-HPPP sample group.

To reveal the functional properties of glycoproteins, the significance level of protein enrichment under functional entries was assessed with the help of GO pathway analysis as an aid to our understanding of the functions of genes and proteins in biological processes and the molecular pathways involved [45]. The results showed that in the HFD versus NC group comparison (Figure 4A), differential glycoproteins were significantly enriched in nuclear transcriptional regulation (e.g., RNA polymerase II-related functional proteins had 53) as well as in nuclear fractions (77% of the total), suggesting that the HFD disrupts intestinal homeostasis by interfering with gene expression; when the HFD versus HFD-PC group comparison was made (Figure 4B), the transcriptional functions remained predominantly transcriptional (e.g., RNA polymerase II regulatory proteins were 44), although the DNA-binding category of molecular functions decreased to 169 (down from 212 in the NC group), suggesting that the HFD-PC group only partially repaired the transcriptional imbalance; a functional remodeling was seen in the comparison of the HFD with the HPPP group (Figure 4C): a dramatic decrease in the number of proteins related to transcriptional regulation (e.g., only 37 in the RNA polymerase II function), and Instead, they were enriched in signaling (101), protein binding (176) and ATP binding (99), reflecting that phytopolysaccharides almost completely blocked the perturbation of glycoprotein function by HFD by strengthening the basic binding and signaling pathways.

**Figure 5:**
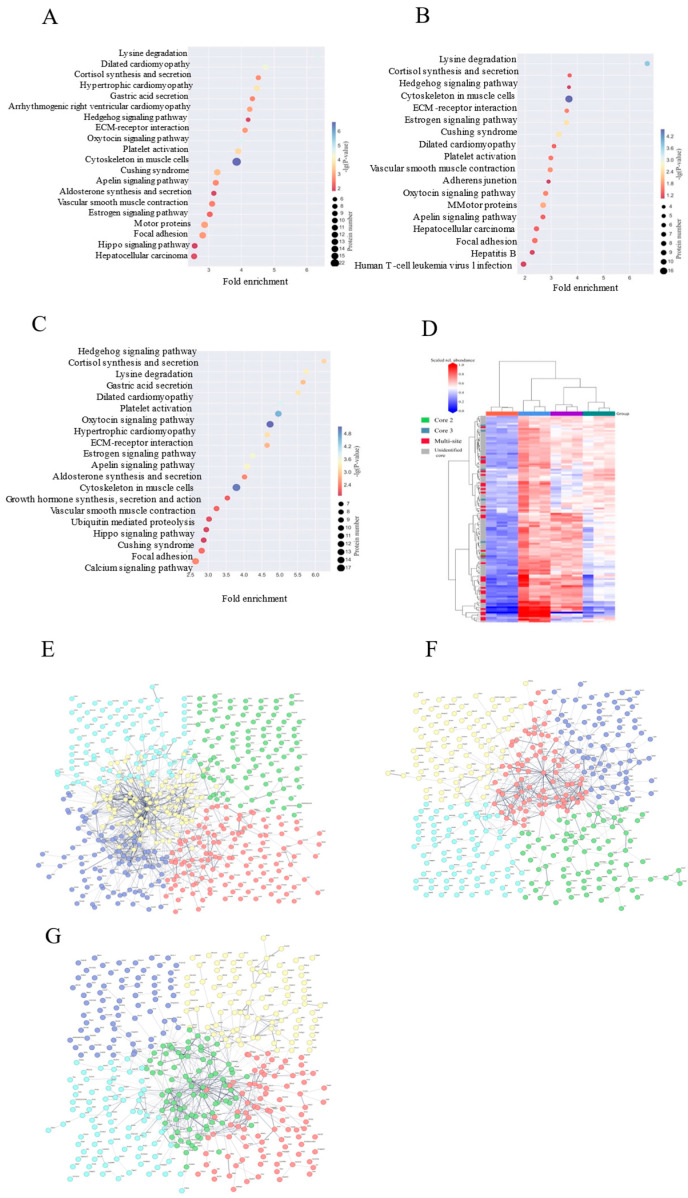
Radical repair of metabolic pathways by HPPP intervention. (**A**) Differential expression of glycoprotein KEGG metabolic pathway analysis in the HFD vs. NC sample group; (**B**) Differential expression of glycoprotein KEGG metabolic pathway analysis in the HFD vs. HFD-PC sample group; (**C**) Differential expression of glycoprotein KEGG metabolic pathway analysis in the HFD vs. HFD-HPPP sample group. (**D**) Heatmap of O-glycosidases found in the colon of mice in each group; (**E**) Differential expression of glycoprotein PPI interactions network in the HFD vs. NC sample group; (**F**) Differential expression of glycoprotein PPI interactions network in the HFD vs. HFD-PC sample group; (**G**) Differential expression of glycoprotein PPI interactions network in the HFD vs. HFD-HPPP sample group.

In addition, it was further revealed by KEGG pathway analysis (Figure 5A–C) that the YD structural domain dominates the disordered N-glycosylation core synthesis, Tox-GHH dom mediates pathogen invasion, and eIF4g_MI induces endoplasmic reticulum stress, which all three constitute the molecular core of the dysfunctional glycosylation. the KEGG pathway confirms that the Hedgehog signaling pathway regulates the cupping cell differentiation via GLI1, while the calcium signaling pathway activates MYLK kinase for barrier disassembly, providing an entry point for targeted intervention.

### 3.9. HPPP Remodeling of the Core 2 O-Glycan Barrier Mitigates HFD-Induced Intestinal Damage

Given that HPPP attenuates HFD-induced intestinal mucosal and alleviates intestinal ecological dysregulation [46], we investigated the mechanisms underlying its therapeutic effects. Since mucosal injury is associated with abnormal changes in mucin O-glycans [47,48], we analyzed the effect of HPPP on the composition of colonic mucin O-glycans in diseased mice using a label-free RPLC-MS/MS technique (Figure 5D) [49]. The results showed that core 2 extended glycan abundance was highest in healthy controls, and its complex branching structure formed a dense barrier by enhancing mucus viscoelasticity to effectively encapsulate commensal flora. The HFD significantly reduced the abundance of core 2 and increased the abundance of aberrant glycosylated structures, and these short or abnormally modified glycans served as carbon sources for pathogenic bacteria (e.g., Enterobacteriaceae) to proliferate and secrete mucinases to accelerate mucus degradation. HPPP restored core 2 abundance and inhibited the generation of aberrant glycans by modulating the glucose metabolism pathway and up-regulating the activity of glycosyltransferases. Repair of mucus layer structure (tight junction proteins claudin-1, occludin, ZO-1, MUC2 expression restored) and remodeling of mucus function.

### 3.10. HPPP Reconstructs Glycoprotein Defense Network

Glycoproteins from colon tissue were analyzed using the STRING tool (version 12.0). Interaction scores with high confidence (0.7) were available, and the thickness of the edges of the network reflected the strength and confidence of the data support (proteins with network breaks open are not indicated). The analysis showed that When HFD was compared with the NC group (Figure 5E), it showed that the HFD triggered a breakdown of glycoprotein interactions: the multicolored nodes were densely clustered and promiscuously connected, corroborating the pre-discovered systemic damage and reflecting the fact that HFD leads to the disordered functional modules—the actin skeleton, and the aberrant interactions of nuclear transcription proteins to form a pathologic network; When HFD was compared with the PC group (Figure 5F), the illustration showed that the drug intervention only partially relieved: nodes were spatially separated and sparsely connected, which alleviated some entanglement but formed functional compensatory silos, and could not re-establish synergistic defenses; whereas, the HPPP achieved radical network remodeling (Figure 5G).

### 3.11. Structure Dependence of High-Fat Diet-Induced O-Glycosylation Inhibition

**Figure 6:**
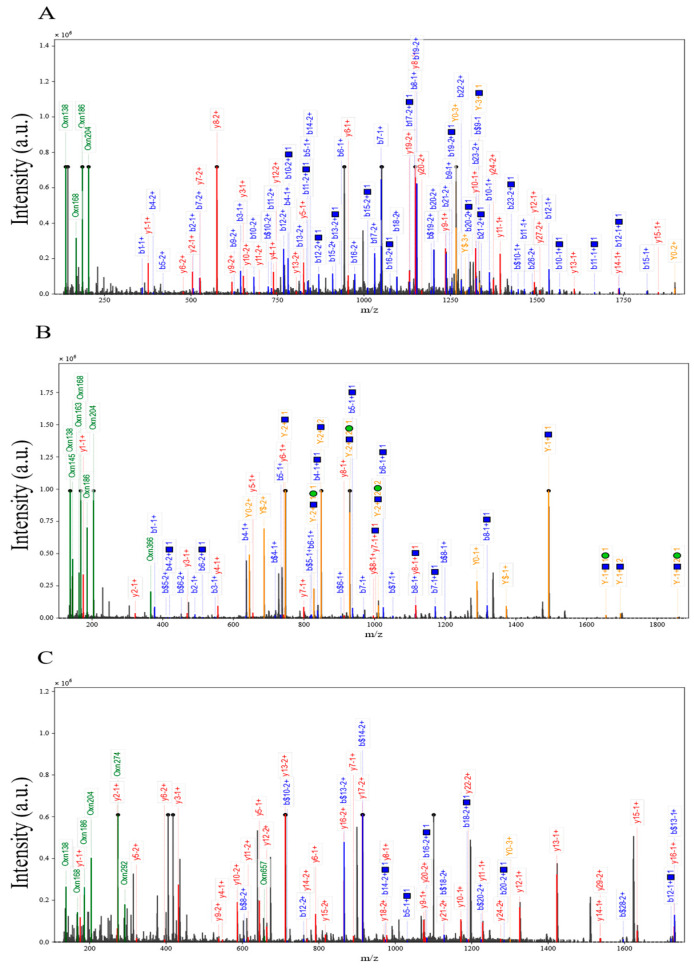
Structure dependence of high-fat diet-induced inhibition of O-glycosylation. (**A**) HFD vs. NC down-regulation of intact O-glycopeptide EFHHGPDPTDTAPGEQDQDVASSPPESS mass spectral dissociation plots; (**B**) HFD vs. HFD-PC down-regulation of intact O-glycopeptide FJGSVSFFR mass spectral dissociation plots; (**C**) HFD vs. HFD-HPPP down-regulation of intact O-glycopeptide PTPRFPQAPEPAEPTDLPPPLPPGPPSVFPDCPR mass spectrometry dissociation plot.

Differences in O-glycopeptide expression between the HFD group and three different control groups were compared by mass spectrometry dissociation analysis. The results clarified that all three specific O-glycopeptides showed a significant down-regulation trend under HFD intervention (Figure 6A–C), and the intensities of the characteristic peaks in their mass spectrometry dissociation profiles were generally lower than those of the control group, suggesting that the glycosylation modification levels might be altered by the effects of lipid metabolism disorders. Specifically, in the EFHHGPDPTDTAPGEQDQDVASSPPESSFQK glycopeptide, the signal intensity of the dissociation peak was significantly weakened (Figure 6A), suggesting that the O-glycosylation modification of this peptide in the core structural domain may be inhibited; the mass spectrometry signals of the FJGSVSFFR glycopeptide showed systematic decreases (Figure 6B), and it was speculated that the HFD may have interfered with the peptide’s glycosylation stability or modification site accessibility; dissociation plots of the glycopeptide in PTPRFPQAPEPAEPTDLPPPLPPGPPSVFPDCPR showed decreased intensity of multiple characteristic peaks at the N-terminal (PTPRFP) and C-terminal (VFPDCPR) ends (Figure 6C), and its characteristic acetylation modification markers (N(1)A(1)) suggested that this post-translational modification may synergize with O- glycosylation involved in lipid metabolism stress response. Taken together, HFD-induced metabolic stress may lead to down-regulation of the abundance of the three O-glycopeptides by interfering with the efficiency of glycosylation enzyme recognition or modification of specific amino acid sequences. Notably, the baseline signal intensity of the PC control group was higher than that of the NC group, suggesting baseline differences in glycopeptide expression by different control models, while the relative abundance of the TR2 peptide was higher than that of the other peptides, suggesting heterogeneity in the sensitivity of different structural O-glycopeptides to HFD. In the future, it is necessary to combine site-specific glycoform identification and functional validation to further reveal the molecular targets of HFD-regulated O-glycosylation.

## 4. Conclusions

HPPP is an acidic heteropolysaccharide extracted from *Hylocereus polyrhizus* processing waste (e.g., fruit skin and pulp residues). Our data suggest that HPPP ameliorates diet-induced obesity and that its mechanism of action is closely related to changes in the production, glycosylation and secretion of the intestinal mucus layer. These changes were confirmed by relative and absolute quantification techniques. Our study reveals potential new targets associated with the mucus layer that may be involved in the prevention of diet-induced obesity. However, the limitations of this study include the lack of direct experimental evidence regarding the biological functions of key glycosylation sites. Therefore, future studies can utilize glycosylation-engineered organoid models to elucidate the regulatory mechanisms of specific sugar chain structures on the spatial distribution of microbiota colonization and determine how these glycosylation sites affect the physicochemical properties of the mucus layer in intestinal epithelial cell lines.

## Figures and Tables

**Table 1:** Yield and compositional analysis of HPPP.

Component	Content (%)
yield	4.4
Total sugar content	16.43 ± 0.35
Galacturonic acid content	10.39 ± 0.90
Protein content	3.51 ± 0.79

**Table 2:** Proteins with differential expression.

Protein	Peptide	PlausibleStruct	GlySite	Type
Dcn	ISDTJITAIPQGLPTSLTEVHLDGNK	(N(H)(N(H(N(H(H(N(H(G)))))))))	2	down
Fbn1	SLDQSGASCEDVDECEGNHR	(N)	1	down
Lamb1	QADEDIQGTQNLLTS	(N)	9	down
Vasp	VTTSEAHPSTPCSSDDSDLER	(N(N))	2	up
Hspg2	EPGYTGQYCEQCAPGYEGDPNVQGGR	(N(G))	5	down

## Data Availability

The original contributions presented in this study are included in the article. Further inquiries can be directed to the corresponding author.

## Abbreviations

The following abbreviations are used in this manuscript:

HPPP	*Hylocereus polyrhizus* pulp residues polysaccharide
HFD	high-fat diet
MetS	Metabolic syndrome
TFA	Trifluoroacetic acid
